# Non-linear association between dietary fiber intake and cognitive function mediated by vitamin E: a cross-sectional study in older adults

**DOI:** 10.3389/fnut.2025.1611162

**Published:** 2025-07-02

**Authors:** Qianyi He, Lucy An, Yue Yue, Can Cui, Chongjian Wang, Hong Xu, Yunfei Guo, Xinyu Zhao

**Affiliations:** ^1^Department of Neurology, The First Affiliated Hospital of Zhengzhou University, Zhengzhou, Henan, China; ^2^Faculty of Medicine and Health Sciences, McGill University, Montreal, QC, Canada; ^3^Department of Epidemiology and Biostatistics, College of Public Health, Zhengzhou University, Zhengzhou, Henan, China; ^4^School of Biomedical Engineering, Shanghai Jiao Tong University, Shanghai, China

**Keywords:** dietary fiber, vitamin E, cognitive function, threshold effect, mediation analysis

## Abstract

**Background:**

Emerging evidence suggests dietary fiber may prevent cognitive decline, but its dose-response relationship and underlying mechanisms remain unclear. This study investigates the non-linear association between dietary fiber intake and cognitive function in older adults and explores the mediating role of vitamin E.

**Methods:**

This cross-sectional analysis of nationally representative National Health and Nutrition Examination Survey (NHANES) Data from 2011 to 2014 included 2,713 adults aged ≥60 years. Dietary fiber intake was assessed using two 24-h dietary recalls. Cognitive function was evaluated using a comprehensive battery comprising three standardized assessments: the Digit Symbol Substitution Test (DSST) to measure processing speed, the Animal Fluency Test (AFT) to assess executive function, and a Consortium to Establish a Registry for Alzheimer's Disease (CERAD) subtest to evaluate memory performance. Composite *z*-scores were calculated for each individual test and combined to generate a global cognition composite score. Generalized additive models (GAM) were applied to model non-linear relationships, and threshold effects were evaluated using two-piece-wise linear regression. Mediation analysis quantified the mediating role of vitamin E in the dietary fiber-cognitive function association, with effects assessed via the non-parametric percentile bootstrap method. Subgroup-specific sensitivity analyses demonstrated consistent findings.

**Results:**

A J-shaped relationship between cognitive function and dietary fiber intake was identified using a two-piece-wise linear regression model. DSST scores reached a plateau at 29.65 g/day of fiber intake (likelihood ratio test *P* < 0.001), while composite *z*-scores reached a plateau at 22.65 g/day (likelihood ratio test *P* = 0.018). Below the inflection point, dietary fiber intake demonstrated a positive association with DSST scores (β: 0.18, 95% CI: 0.01–0.26, *P* < 0.0001), whereas above this threshold, the relationship became negative (β: −0.15, 95% CI: −0.29 to −0.02, *P* = 0.0265). Similarly, for composite *z*-scores, a positive association was observed below the inflection point (β: 0.01, 95% CI: 0.00–0.01, *P* = 0.0004), while the relationship appeared to saturate above this threshold (β: −0.00, 95% CI: −0.01–0.00, *P* = 0.9043). Mediation analysis revealed that vitamin E intake significantly mediated 85.0% (*P* < 0.0001) of the association between dietary fiber intake and composite *z*-scores, and 86.8% (*P* < 0.0001) of the association between dietary fiber intake and DSST scores.

**Conclusion:**

Moderate dietary fiber intake is associated with optimal cognitive performance, largely mediated by vitamin E.

## 1 Introduction

Cognitive decline in aging populations poses significant challenges to both public health systems and the quality of life of older adults globally. As the population ages, the incidence of neurodegenerative diseases such as Alzheimer's disease (AD) increases (1), thus the urgency for more effective preventive strategies. Among various factors, dietary interventions have gained attention for their potential neuroprotective effects (2, 3). Recent investigations have particularly highlighted the role of dietary fiber, which has been associated with improved cognitive function in older adults (4, 5). However, current studies primarily examine linear relationships, often neglecting the potential for non-linear interactions, including possible threshold effects in nutrient benefits.

Recent evidence suggests that while increasing dietary fiber intake can enhance cognitive function, the benefits may plateau at moderate levels of intake. For instance, a study by Li et al. (4) identified a significant correlation between dietary fiber consumption and cognitive performance, suggesting that consumption of ~34 g of fiber per day maximizes cognitive benefits. These findings align with another study noting the prevalence of low dietary fiber intake worldwide, with many populations not meeting recommended levels of fiber intake (6). Thus, understanding the optimal intake of dietary fiber and its implications for cognitive function warrants further exploration, particularly under the hypothesis of a non-linear J-shaped relationship, where cognitive improvements occur at moderate levels and potential adverse effects may arise from both extremes.

In addition to dietary fiber, emerging evidence supports the mediating role of nutrients such as vitamin E—an antioxidant linked to cognitive health by reducing oxidative stress which is a significant contributor to neurodegenerative diseases (7, 8). A systematic review indicated that increased vitamin E intake, particularly from natural sources, correlates with better cognitive performance and may protect against cognitive decline (7). Furthermore, the relationship between these dietary components and neuronal health may be influenced by their capacity to modulate inflammation and preserve synaptic integrity, thereby contributing to neuroprotection (9).

This study aims to address gaps in the literature by investigating the non-linear relationship between dietary fiber intake and cognitive function in older adults. Compared to traditional linear models, non-linear curve fitting offers greater flexibility in capturing the complex relationships present in real-world data, thereby enhancing the adaptability and interpretability of scientific analyses (10, 11). In biomedical and clinical research, the use of curve fitting enables a more accurate depiction of physiological processes, reduces fitting errors, and improves the reliability of research findings (11, 12), providing a scientific basis for recommending dietary fiber intake levels in the elderly population. Furthermore, this study explores the mediating role of vitamin E in the relationship between dietary fiber intake and cognitive function.

To achieve this, data from the National Health and Nutrition Examination Survey (NHANES) of the 2011–2014 cohort provides a robust analytical framework, allowing for detailed assessment of cognitive function relative to varying levels of dietary fiber and vitamin E intake. We hypothesized that dietary fiber's effect on cognitive function follows a non-linear pattern with a potential threshold effect, and that this relationship is significantly mediated by vitamin E.

## 2 Methods

### 2.1 Data sources and study population

This cross-sectional study followed the Strengthening the Reporting of Observational Studies in Epidemiology (STROBE) guidelines. Data were obtained from the NHANES 2011–2014 cycles. NHANES employs a complex, multistage probability sampling design to select participants representative of the non-institutionalized US civilian population. Data collection conducted by trained personnel following standardized protocols. The survey protocol was approved by the National Center for Health Statistics (NCHS) Research Ethics Committee, and all participants provided written informed consent prior to data collection.

**Figure 1:**
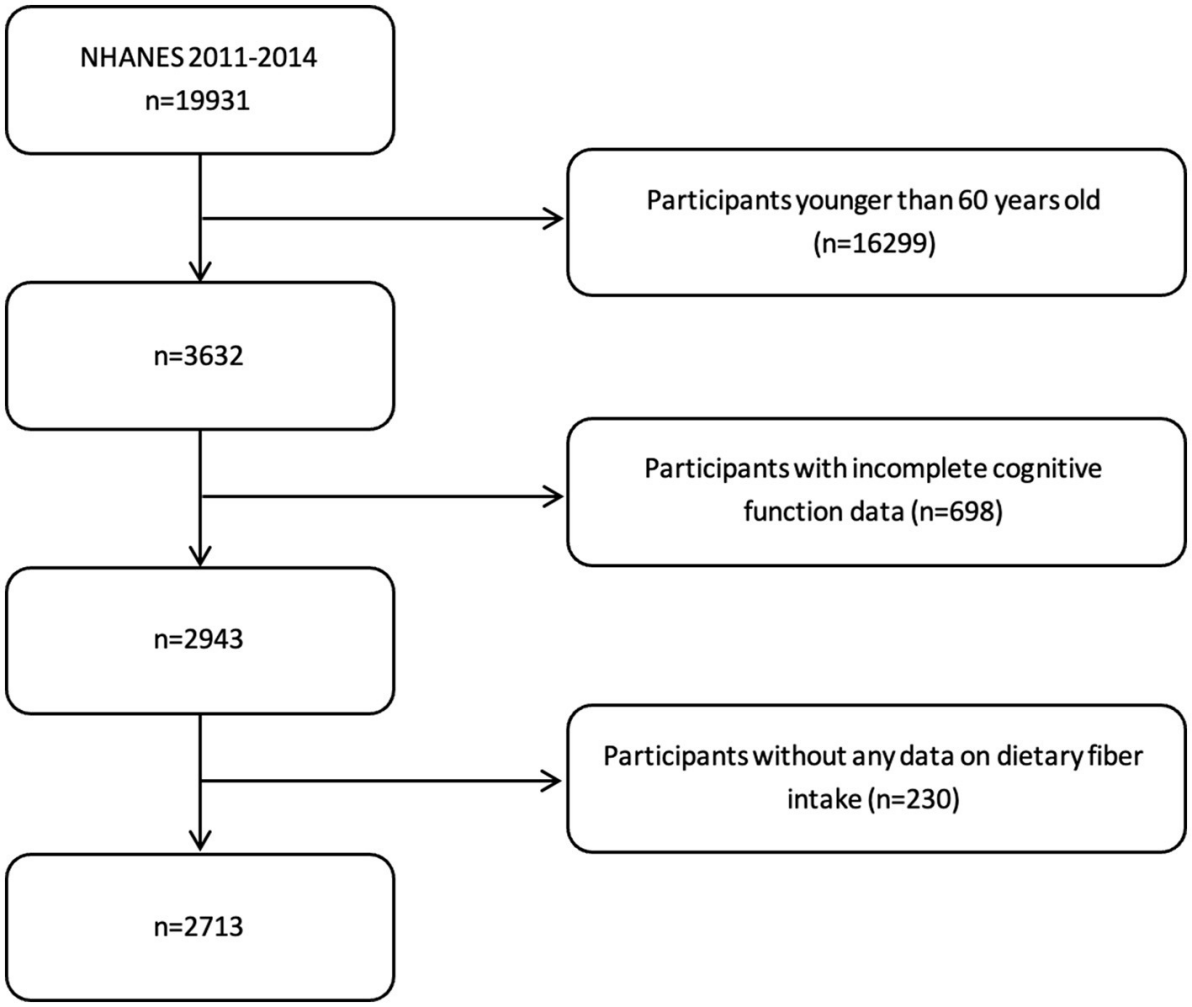
Participant selection and data collection process.

Study participants underwent comprehensive health interviews that collected sociodemographic information (including age, gender, race/ethnicity, education level, marital status, and annual family income) and health-related behavioral data (encompassing alcohol consumption, smoking status, and physical activity levels). The interview also gathered information about medical conditions such as hypertension, diabetes, and depression, along with anthropometric measurements including body mass index (BMI) and waist circumference. From the initial sample, we included participants at or over the age of 60 and excluded those with incomplete cognitive assessment data and those with missing dietary fiber intake information. The final analytical sample comprised 2,713 adults (Figure 1).

### 2.2 Dietary assessment

Total dietary fiber intake (g/day) was the primary exposure in this study, while vitamin E intake (mg/day) was investigated as a potential mediator. Dietary data were collected through two 24-h dietary recalls, with the first recall conducted in-person and the second recall completed via telephone 3–10 days later. All dietary interviews were conducted by trained dietary interviewers using the United States Department of Agriculture's Automated Multiple-Pass Method, which employs a five-step process to enhance the accuracy and completeness of dietary recall. Nutrient intake calculations were performed using the United States Department of Agriculture's Food and Nutrient Database for Dietary Studies. Daily nutrient intakes were calculated by averaging the values from these two 24-h dietary recalls.

### 2.3 Cognitive function assessment

Cognitive function was evaluated using four standardized neuropsychological tests and served as the outcome measure of this study. The Consortium to Establish a Registry for Alzheimer's Disease (CERAD) Word Learning Test was administered to assess verbal learning and memory (13). This test consisted of two components: the immediate recall test (CERAD.IRT) and the delayed recall test (CERAD.DRT). For the immediate recall, participants were asked to read aloud and recall a list of 10 unrelated words, with the sum of correct responses from three consecutive trials recorded. The delayed recall was conducted 8–10 min after the initial learning trials, with scores ranging from 0 to 10. Executive function was assessed using the Animal Fluency Test (AFT), which required participants to name as many animals as possible within 1 min, with one point awarded for each correct animal named (14, 15). Processing speed and executive function were evaluated using the Digit Symbol Substitution Test (DSST) (16), where participants were given 2 min to match symbols to numbers in 133 boxes according to a provided legend, with scores ranging from 0 to 133 based on correct matches.

Global cognitive function was quantified using a composite *z*-score, calculated by averaging the standardized scores of all four cognitive tests (DSST, AFT, CERAD.IRT, and CERAD.DRT). Each test score was standardized based on the sample mean and standard deviation (SD), with higher scores indicating better cognitive performance (15, 17).

### 2.4 Covariates

Covariates were selected based on their potential confounding effects in the relationship between dietary fiber intake and cognitive performance. Age (years), gender (male, female), race (Mexican American, other Hispanic, non-Hispanic White, non-Hispanic Black, non-Hispanic Asian, and other racial groups), annual family income (<20,000, 20,000–75,000, and >75,000 dollars), education level (<9, 9–12, or >12 years), marital status (married/living with partner or living alone), and medical history (history of hypertension, diabetes, and depression) were examined as demographic and health-related covariates. Anthropometric and lifestyle factors including BMI (kg/m^2^), waist circumference (cm), daily energy intake (kcal), alcohol consumption, smoking status, physical activity, and dietary vitamin intake (vitamins B1, B2, B6, B12, C, D, and E) were also considered as potential confounders.

Physical activity was quantified using total metabolic equivalent (MET) minutes of moderate to vigorous work and leisure activities, calculated from NHANES MET scores by multiplying the frequency, duration, and intensity. A threshold of 600 MET minutes per week was used to distinguish between high and low activity levels, with high activity levels offering significant health benefits (18). Smoking status was categorized into three groups: never smokers (lifetime consumption of fewer than 100 cigarettes), current smokers, and former smokers (those who quit after smoking more than 100 cigarettes). Similarly, alcohol consumption was classified into three groups: never drinkers (lifetime consumption of fewer than 12 drinks), current drinkers, and former drinkers (those who quit after consuming at least 12 drinks in their lifetime) (19).

### 2.5 Statistical analysis

Statistical analyses were performed using EmpowerStats (https://www.empowerstats.net/en/, X&Y Solutions, Inc., Boston, MA, USA) and R software version 4.4.1. A two-sided *P*-value <0.05 was considered to be statistically significant. Continuous variables with normal distribution were expressed as mean ± standard deviation (SD), while those with skewed distribution were presented as median with interquartile range. Categorical variables were described as frequencies and percentages. Participants were categorized into quartiles based on dietary fiber intake (Q1, ≤ 10.9 g/day; Q2, 10.9–15.4 g/day; Q3, 15.4–21.2 g/day; Q4, >21.2 g/day). In between-group comparisons were conducted using one-way analysis of variance (ANOVA) for normally distributed continuous variables, Kruskal–Wallis *H* test for skewed continuous variables, and chi-squared tests for categorical variables.

Associations between dietary fiber intake and cognitive performance were examined using univariate and multivariate linear regression models. Results are presented as β coefficients with 95% confidence intervals (CI). Potential confounders were selected based on their associations with outcomes of interest or a change in effect estimates exceeding 10% (20). Multicollinearity was assessed using the variance inflation factor (VIF), with VIF ≤ 5 indicating acceptable collinearity levels (Supplementary Table S1) (21). Variables demonstrating multicollinearity with cognitive function scores and dietary fiber (VIF > 5) were excluded from analysis. The multivariate models were adjusted for gender, age, race, education level, annual family income, alcohol status, hypertension, diabetes, physical activity, depression, and intake of vitamins B1 and D.

To investigate potential non-linear relationships between dietary fiber intake and cognitive performance, we employed generalized additive models (GAM) with smooth curve fitting. Two-piece-wise linear regression models were used to examine threshold effects, and the optimal threshold was determined using a log-likelihood ratio test comparing one-line linear regression with two-piece-wise linear models. The 95% CI for the turning point was calculated using bootstrap resampling.

Mediation analysis was performed using the “mediation” package in R version 4.4.1 to assess whether vitamin E intake mediated the association between dietary fiber intake and cognitive performance. Models were adjusted for the previously mentioned covariates. A significant mediating effect was established when all of the following criteria were met: significant indirect effect, significant total effect, and positive proportion of mediator effect. Mediation effects were assessed using the non-parametric percentile bootstrap method with 1,000 resamples to generate 95% confidence intervals. Mediation was considered statistically significant when confidence intervals excluded zero. To test the robustness of analysis, we performed two-piece-wise linear regression and mediation analysis stratified by gender (male, female), hypertension (yes or no), diabetes (yes, no, or borderline), and depression (yes or no). Several steps were taken to address potential sources of bias: (1) selection bias: NHANES sampling weights were applied to maintain national representativeness; (2) measurement bias: standardized assessment protocols were followed by trained personnel; (3) recall bias: the Automated Multiple-Pass Method was used to improve dietary recall accuracy; and (4) confounding: comprehensive adjustment for sociodemographic, lifestyle, and health factors.

## 3 Results

### 3.1 Characteristics of participants at the baseline

This cross-sectional study included 2,713 participants with a mean age of 69.4 ± 6.8 years (Table 1), categorized into four quartiles based on dietary fiber intake. Higher fiber intake was significantly associated with male gender, higher education level, better socioeconomic status, and healthier lifestyle behaviors including lower smoking rates and increased physical activity (all *P* < 0.001). Additionally, participants in the highest fiber intake quartile (Q4) demonstrated lower prevalence of hypertension (57.2 vs. 67.9%, *P* < 0.001) and diabetes (19.9 vs. 27.5%, *P* < 0.001) compared to the lowest quartile (Q1). The DSST scores showed a clear positive trend across quartiles, with Q4 participants achieving notably higher scores (49.0 ± 16.6) compared to Q1 (42.2 ± 17.4, *P* < 0.001). Similarly, the AFT results exhibited a gradual improvement from Q1 (16.8 ± 5.6) to Q4 (18.2 ± 5.7, *P* < 0.001). The composite *Z*-scores revealed a significant positive association with fiber intake, progressing from −0.2 ± 0.8 in Q1 to 0.1 ± 0.7 in Q4 (*P* < 0.001).

**Table 1:** Baseline characteristics of the study population according to dietary fiber quartiles.

**Variables**	**Dietary fiber, g/day**					***P*-value**
	**Total**	**Q1 (0.2–10.9)**	**Q2 (10.9–15.4)**	**Q3 (15.4–21.2)**	**Q4 (21.3–118.4)**	
Number of participants	2,713	674	682	675	682	
Age, (years)	69.4 ± 6.8	69.2 ± 6.7	69.7 ± 6.8	69.8 ± 6.9	69.0 ± 6.6	0.087
**Gender**, ***n*** **(%)**						<0.001
Male	1,336 (49.2)	281 (41.7)	310 (45.5)	334 (49.5)	411 (60.3)	
Female	1,377 (50.8)	393 (58.3)	372 (54.5)	341 (50.5)	271 (39.7)	
**Race/ethnicity**, ***n*** **(%)**						<0.001
Mexican American	233 (8.6)	37 (5.5)	53 (7.8)	54 (8.0)	89 (13.0)	
Other Hispanic	274 (10.1)	69 (10.2)	66 (9.7)	74 (11.0)	65 (9.5)	
Non-Hispanic White	1,332 (49.1)	303 (45.0)	343 (50.3)	356 (52.7)	330 (48.4)	
Non-Hispanic Black	642 (23.7)	221 (32.8)	173 (25.4)	130 (19.3)	118 (17.3)	
Other race	232 (8.6)	44 (6.5)	47 (6.9)	61 (9.0)	80 (11.7)	
**Education level**, ***n*** **(%)**						<0.001
<9 years	294 (10.8)	90 (13.4)	74 (10.9)	55 (8.2)	75 (11.0)	
9–12 years	1,018 (37.6)	322 (47.8)	268 (39.4)	242 (35.9)	186 (27.3)	
>12 years	1,399 (51.6)	262 (38.9)	338 (49.7)	378 (56.0)	421 (61.7)	
**Marital status**, ***n*** **(%)**						<0.001
Married or Living with partner	1,574 (58.1)	346 (51.5)	383 (56.2)	410 (60.8)	435 (63.8)	
Living alone	1,136 (41.9)	326 (48.5)	299 (43.8)	264 (39.2)	247 (36.2)	
**Annual family income (dollars)**, ***n*** **(%)**						<0.001
<20,000	624 (24.0)	205 (31.7)	158 (24.1)	139 (21.5)	122 (18.6)	
20,000–75,000	1,285 (49.3)	329 (50.9)	326 (49.6)	306 (47.4)	324 (49.5)	
>75,000	696 (26.7)	113 (17.5)	173 (26.3)	201 (31.1)	209 (31.9)	
**Alcohol consumption**, ***n*** **(%)**						0.031
Never	405 (15.1)	111 (16.7)	97 (14.3)	100 (14.9)	97 (14.3)	
Former	761 (28.3)	214 (32.2)	197 (29.1)	179 (26.6)	171 (25.3)	
Current	1,525 (56.7)	340 (51.1)	383 (56.6)	393 (58.5)	409 (60.4)	
**Smoking status**, ***n*** **(%)**						<0.001
Never	1,326 (48.9)	298 (44.3)	337 (49.4)	342 (50.7)	349 (51.3)	
Former	1,045 (38.6)	236 (35.1)	261 (38.3)	262 (38.8)	286 (42.0)	
Current	340 (12.5)	139 (20.7)	84 (12.3)	71 (10.5)	46 (6.8)	
**Physical activity**, ***n*** **(%)**						<0.001
Low physical activity	1,130 (41.7)	345 (51.3)	298 (43.7)	250 (37.1)	237 (34.8)	
High physical activity	1,581 (58.3)	328 (48.7)	384 (56.3)	424 (62.9)	445 (65.3)	
**Hypertension**, ***n*** **(%)**						<0.001
Yes	1,691 (62.4)	456 (67.9)	440 (64.5)	406 (60.2)	389 (57.2)	
No	1,017 (37.6)	216 (32.1)	242 (35.5)	268 (39.8)	291 (42.8)	
**Diabetes**, ***n*** **(%)**						0.015
Yes	633 (23.3)	185 (27.5)	165 (24.2)	150 (22.2)	133 (19.9)	
No	1,954 (72.1)	463 (68.9)	483 (70.8)	488 (72.3)	520 (76.3)	
Borderline	124 (4.6)	24 (3.6)	34 (5.0)	37 (5.5)	29 (4.3)	
Yes	236 (8.8)	69 (10.4)	66 (9.8)	45 (6.7)	56 (8.3)	
No	2,446 (91.2)	593 (89.6)	609 (90.2)	625 (93.3)	619 (91.7)	
BMI, kg/m^2^	29.1 ± 6.4	29.7 ± 7.2	29.6 ± 6.4	28.8 ± 5.9	28.4 ± 5.9	<0.001
Waist circumference, cm	102.1 ± 14.6	102.9 ± 15.3	103.0 ± 14.6	101.3 ± 14.1	101.1 ± 14.4	0.023
**Dietary intake**						
Energy, kcal/day	1,815.7 ± 689.2	1,313.6 ± 507.3	1,697.1 ± 521.8	1,924.6 ± 558.6	2,322.8 ± 723.6	<0.001
Protein, g/day	72.0 ± 29.9	54.3 ± 23.5	68.5 ± 24.7	76.6 ± 26.0	90.9 ± 32.1	<0.001
Fat, g/day	69.3 ± 33.3	50.8 ± 24.8	65.3 ± 26.9	74.0 ± 30.9	86.9 ± 38.0	<0.001
Carbohydrate, g/day	221.7 ± 88.6	152.6 ± 62.6	202.0 ± 63.0	236.1 ± 69.1	295.6 ± 89.2	<0.001
Sugars, g/day	94.9 ± 51.6	69.6 ± 42.3	89.3 ± 46.1	100.3 ± 46.7	120.4 ± 56.6	<0.001
Vitamin B1, mg/day	1.5 ± 0.6	1.0 ± 0.4	1.3 ± 0.5	1.6 ± 0.6	1.9 ± 0.7	<0.001
Vitamin B2, mg/day	1.8 (1.3–2.3)	1.3 (0.9–1.8)	1.7 (1.3–2.1)	1.9 (1.5–2.4)	2.3 (1.8–3.0)	<0.001
Vitamin B6, mg/day	1.7 (1.3–2.3)	1.2 (0.8–1.6)	1.6 (1.2–2.0)	1.9 (1.5–2.4)	2.4 (1.9–3.1)	<0.001
Vitamin B12, μg/day	3.7 (2.4–5.7)	2.9 (1.7–4.4)	3.5 (2.4–5.2)	4.2 (2.8–5.9)	4.6 (2.9–7.0)	<0.001
Vitamin C, mg/day	85.4 ± 80.4	48.9 ± 51.0	75.6 ± 62.1	91.3 ± 63.1	125.5 ± 110.9	<0.001
Vitamin D, μg/day	3.6 (2.0–6.1)	2.6 (1.3–4.5)	3.4 (1.9–5.5)	4.0 (2.2–6.5)	4.8 (2.8–7.8)	<0.001
Vitamin E, mg/day	6.9 (4.7–9.8)	4.3 (2.9–6.1)	6.4 (4.7–8.4)	7.9 (5.8–10.3)	10.0 (7.3–13.7)	<0.001
**Cognitive function**						0.001
CERAD1	4.7 ± 1.7	4.5 ± 1.7	4.8 ± 1.8	4.8 ± 1.7	4.8 ± 1.6	
CERAD2	6.7 ± 1.8	6.5 ± 1.9	6.7 ± 1.8	6.8 ± 1.8	6.9 ± 1.8	<0.001
CERAD3	7.5 ± 1.8	7.4 ± 1.9	7.6 ± 1.8	7.6 ± 1.7	7.6 ± 1.7	0.009
CERAD-IRT	19.0 ± 4.6	18.3 ± 4.8	19.1 ± 4.7	19.2 ± 4.5	19.3 ± 4.4	<0.001
CERAD-DRT	6.0 ± 2.3	5.7 ± 2.4	6.0 ± 2.3	6.1 ± 2.3	6.1 ± 2.2	0.001
AFT	16.7 ± 5.5	15.5 ± 5.3	16.6 ± 5.4	17.0 ± 5.5	17.7 ± 5.5	<0.001
DSST	46.2 ± 17.2	42.2 ± 17.4	45.8 ± 17.0	47.7 ± 17.0	49.0 ± 16.6	<0.001
*Z*-score	0.0 ± 0.8	−0.2 ± 0.8	0.0 ± 0.8	0.1 ± 0.8	0.1 ± 0.7	<0.001

BMI, body mass index; CERAD, Consortium to Establish a Registry for Alzheimer's Disease; IRT, Item Response Theory; DRT, Delayed Recall Test; AFT, Animal Fluency Test; DSST, Digit Symbol Substitution Test; z-score, standardized composite cognitive score.

### 3.2 Multiple linear regression analysis of dietary fiber intake and cognitive performance

Multiple linear regression models were employed to investigate the association between dietary fiber intake quartiles and cognitive performance (Table 2). After comprehensive adjustment for potential confounders in the Adjust II model (including race, education level, annual family income, alcohol status, hypertension, diabetes, physical activity, depression, vitamin B1 intake, and vitamin D intake), the associations with CERAD.IRT and CERAD.DRT were attenuated and lost statistical significance (all *P* values >0.05). Most notably, both DSST and the composite *z*-score demonstrated consistent associations with dietary fiber intake across all quartiles compared to the reference group (Q1). For DSST, statistically significant improvements were observed across all higher quartiles: Q2 (β = 1.60, 95% CI: 0.22–2.97, *P* = 0.0230), Q3 (β = 1.84, 95% CI: 0.40–3.29, *P* = 0.0123), and Q4 (β = 2.76, 95% CI: 1.18–4.34, *P* = 0.0006). Similarly, the composite *z*-score showed consistent positive associations across all fiber intake quartiles: Q2 (β = 0.08, 95% CI: 0.01–0.15, *P* = 0.0221), Q3 (β = 0.09, 95% CI: 0.01–0.16, *P* = 0.0186), and Q4 (β = 0.12, 95% CI: 0.04–0.20, *P* = 0.0029). In contrast, AFT showed statistical significance only in the highest quartile (Q4: β = 0.76, 95% CI: 0.14–1.39, *P* = 0.0162), with Q2 and Q3 failing to reach significance (*P* = 0.1159 and *P* = 0.0956, respectively).

**Table 2:** Association between dietary fiber intake quartiles and cognitive performance measures in multiple regression models.

**Exposure**	**Non-adjusted β (95% CI)**	***P*-value**	**Adjust I β (95% CI)**	***P*-value**	**Adjust II β (95% CI)**	***P*-value**
**CERAD.IRT**						
Dietary fiber intake Q1	Reference	–	Reference	–	Reference	–
Dietary fiber intake Q2	0.73 (0.24, 1.22)	0.0034	0.88 (0.42, 1.35)	0.0002	0.37 (−0.10, 0.83)	0.1223
Dietary fiber intake Q3	0.90 (0.41, 1.39)	0.0004	1.14 (0.67, 1.61)	<0.0001	0.39 (−0.10, 0.88)	0.1152
Dietary fiber intake Q4	0.99 (0.50, 1.48)	<0.0001	1.28 (0.82, 1.75)	<0.0001	0.45 (−0.09,0.98)	0.1007
**CERAD.DRT**						
Dietary fiber intake Q1	Reference	–	Reference	–	Reference	–
Dietary fiber intake Q2	0.31 (0.07, 0.56)	0.0127	0.39 (0.16, 0.62)	0.0010	0.16 (−0.08, 0.39)	0.1894
Dietary fiber intake Q3	0.39 (0.15, 0.64)	0.0017	0.52 (0.28, 0.75)	<0.0001	0.15 (−0.10, 0.39)	0.2356
Dietary fiber intake Q4	0.47 (0.22, 0.71)	0.0002	0.61 (0.37, 0.84)	<0.0001	0.18 (−0.08, 0.45)	0.1772
**AFT**						
Dietary fiber intake Q1	Reference	–	Reference	–	Reference	–
Dietary fiber intake Q2	1.11 (0.53, 1.69)	0.0002	1.19 (0.63, 1.76)	<0.0001	0.43 (−0.11, 0.98)	0.1159
Dietary fiber intake Q3	1.53 (0.95, 2.11)	<0.0001	1.63 (1.06, 2.20)	<0.0001	0.48 (−0.08, 1.05)	0.0956
Dietary fiber intake Q4	2.20 (1.62, 2.78)	<0.0001	2.15 (1.58, 2.72)	<0.0001	0.76 (0.14, 1.39)	0.0162
**DSST**						
Dietary fiber intake Q1	Reference	–	Reference	–	Reference	–
Dietary fiber intake Q2	3.65 (1.84, 5.46)	<0.0001	4.21 (2.49, 5.92)	<0.0001	1.60 (0.22, 2.97)	0.0230
Dietary fiber intake Q3	5.51 (3.70, 7.33)	<0.0001	6.39 (4.67, 8.11)	<0.0001	1.84 (0.40, 3.29)	0.0123
Dietary fiber intake Q4	6.83 (5.02, 8.64)	<0.0001	7.77 (6.04, 9.49)	<0.0001	2.76 (1.18, 4.34)	0.0006
***Z*** **-score**						
Dietary fiber intake Q1	Reference	–	Reference	–	Reference	–
Dietary fiber intake Q2	0.18 (0.09, 0.26)	<0.0001	0.20 (0.13, 0.28)	<0.0001	0.08 (0.01, 0.15)	0.0221
Dietary fiber intake Q3	0.24 (0.16, 0.32)	<0.0001	0.28 (0.21, 0.36)	<0.0001	0.09 (0.01, 0.16)	0.0186
Dietary fiber intake Q4	0.30 (0.22, 0.38)	<0.0001	0.34 (0.27, 0.42)	<0.0001	0.12 (0.04, 0.20)	0.0029

CERAD.IRT, Consortium to Establish a Registry for Alzheimer's Disease Immediate Recall Test; CERAD.DRT, CERAD Delayed Recall Test; AFT, Animal Fluency Test; DSST, Digit Symbol Substitution Test; z-score, Standardized composite cognitive score; Q1–Q4, Quartiles of dietary fiber intake (Q1 lowest, Q4 highest).

Model adjustments: Non-adjusted: No covariates. Adjust I: Adjusted for gender and age. Adjust II: Adjusted for gender, age, race, education level, annual family income, alcohol status, hypertension, diabetes, physical activity, depression, vitamin B1 intake, and vitamin D intake.

### 3.3 Threshold effect analysis of dietary fiber intake on cognitive function

**Figure 2:**
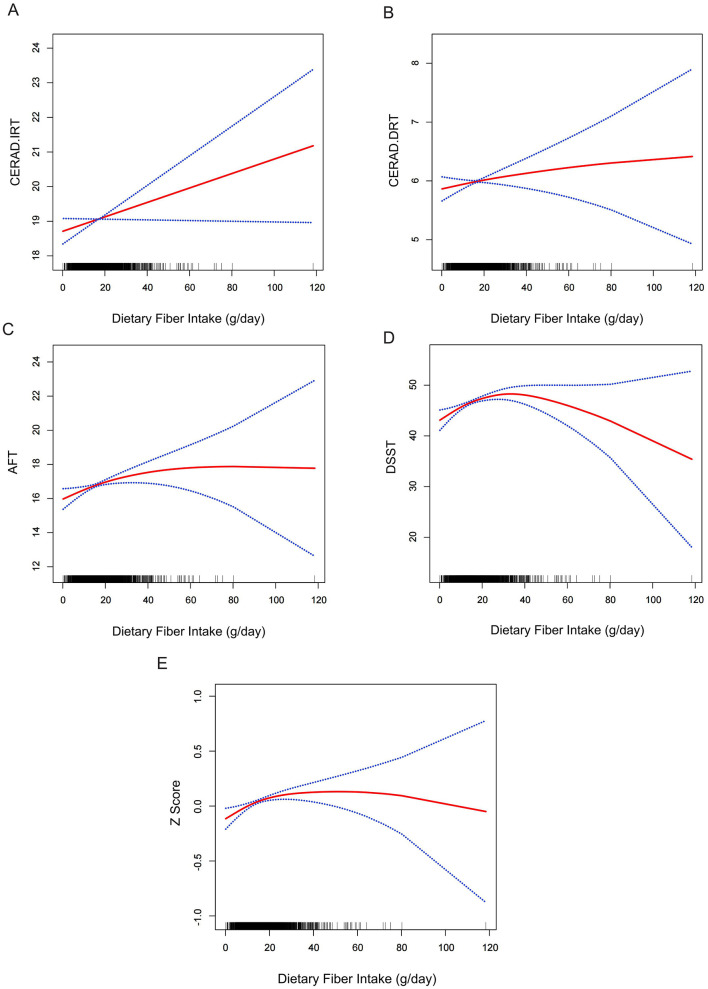
Relationship between dietary fiber intake and cognitive performance measures. General additive models (GAM) illustrating the association between dietary fiber intake (g) and cognitive function across multiple cognitive assessments: **(A)** Consortium to Establish a Registry for Alzheimer's Disease Immediate Recall Test (CERAD.IRT) scores, **(B)** CERAD Delayed Recall Test (CERAD.DRT) scores, **(C)** Animal Fluency Test (AFT) scores, **(D)** Digit Symbol Substitution Test (DSST) performance, and **(E)** Composite *z*-score representing overall cognitive performance. The *x*-axis represents dietary fiber intake (g), while the *y*-axis displays the standardized cognitive performance scores. The solid red line indicates the smooth curve fit between variables, with blue bands representing the 95% confidence intervals. All models were adjusted for demographic and health-related covariates including gender, age, race, education level, annual family income, alcohol consumption status, hypertension, diabetes, physical activity, BMI, energy intake, depression, and vitamin B1 and D intake levels (*n* = 2,529).

Two-piece-wise linear regression analysis revealed varying relationships between dietary fiber intake and cognitive function across different cognitive assessments (Table 3). The DSST scores demonstrated a significant non-linear association with an inflection point at 29.65 g/day of fiber intake (likelihood ratio test *P* < 0.001), while the composite *z*-score showed a threshold at 22.65 g/day (likelihood ratio test *P* = 0.018). Below threshold, dietary fiber intake exhibited a positive association with DSST (β: 0.18, 95% CI: 0.01–0.26, *P* < 0.0001), whereas above this threshold, the relationship became negative (β: −0.15, 95% CI: −0.29 to −0.02, *P* = 0.0265). Similarly, the composite *z*-score showed a positive correlation below its inflection point (β: 0.01, 95% CI: 0.00–0.01, *P* = 0.0004), but the association plateaued past this threshold (β: −0.00, 95% CI: −0.01–0.00, *P* = 0.9043). In contrast, the AFT (likelihood ratio test *P* = 0.078), CERAD.IRT (likelihood ratio test *P* = 0.213) and CERAD.DRT (likelihood ratio test *P* = 0.220) did not demonstrate significant non-linear relationships. Then we used a GAM with smooth curve fitting (Figure 2). All models were adjusted for gender, age, race, education level, household annual income, drinking status, hypertension, diabetes, physical activity, depression, and intake of vitamins B1 and D.

**Table 3:** Threshold effect analysis of the association between dietary fiber intake and cognitive function measures (total *n* = 2,529).

**Outcome**	**CERAD.IRT β (95% CI)**	***P*-value**	**CERAD.DRT β (95% CI)**	***P*-value**	**AFT β (95% CI)**	***P*-value**	**DSST β (95% CI)**	***P*-value**	***Z*-score β (95% CI)**	***P*-value**
**Model I**										
One line effect	0.02 (−0.00, 0.04)	0.0615	0.01 (−0.01, 0.02)	0.2646	0.03 (0.01, 0.06)	0.0097	0.08 (0.01, 0.14)	0.0182	0.00 (0.00, 0.01)	0.0064
**Model II**										
Turning Point (K)	18.97	–	18.97	–	20.16	–	29.65	–	22.65	–
Dietary fiber intake <K	0.04 (0.00, 0.09)	0.0426	0.02(−0.00, 0.04)	0.1048	0.07 (0.02, 0.11)	0.0038	0.18 (0.10, 0.26)	<0.0001	0.01 (0.00, 0.01)	0.0004
Dietary fiber intake ≥ K	0.01 (−0.02, 0.04)	0.6247	−0.00 (−0.02, 0.01)	0.9664	0.01 (−0.03, 0.05)	0.6060	−0.15 (−0.29, −0.02)	0.0265	−0.00 (−0.01, 0.00)	0.9043
*P* value for LRT test	–	0.213	–	0.220	–	0.078	–	<0.001	–	0.018
95% CI for tuning point	19.11–19.70		6.00–6.29		17.24–17.96		50.47–53.48		0.13–0.24	

CERAD.IRT, Consortium to Establish a Registry for Alzheimer's Disease Immediate Recall Test; CERAD.DRT, Consortium to Establish a Registry for Alzheimer's Disease Delayed Recall Test; AFT, Animal Fluency Test; DSST, Digit Symbol Substitution Test; z-score, Composite cognitive score; LRT, logarithm likelihood ratio test.

Model adjustments: Model I represents linear regression analysis; Model II represents curve-fitting threshold effect analysis. All models were adjusted for gender, age, race, education level, annual family income, alcohol status, hypertension, diabetes, physical activity, depression, vitamin B1 intake, and vitamin D intake.

Subgroup analyses stratified by gender, hypertension status, depression status, and diabetes categorization (including borderline cases) revealed significant threshold effects between dietary fiber intake and both DSST scores (Supplementary Tables S2–S5) and composite *z*-scores (Supplementary Tables S6–S9). Notably, these non-linear associations were consistently observed across all examined subgroups, with no significant interaction effects detected (all *P*-interaction >0.05).

### 3.4 Mediation analysis of vitamin E and dietary fiber on cognitive function

**Figure 3:**
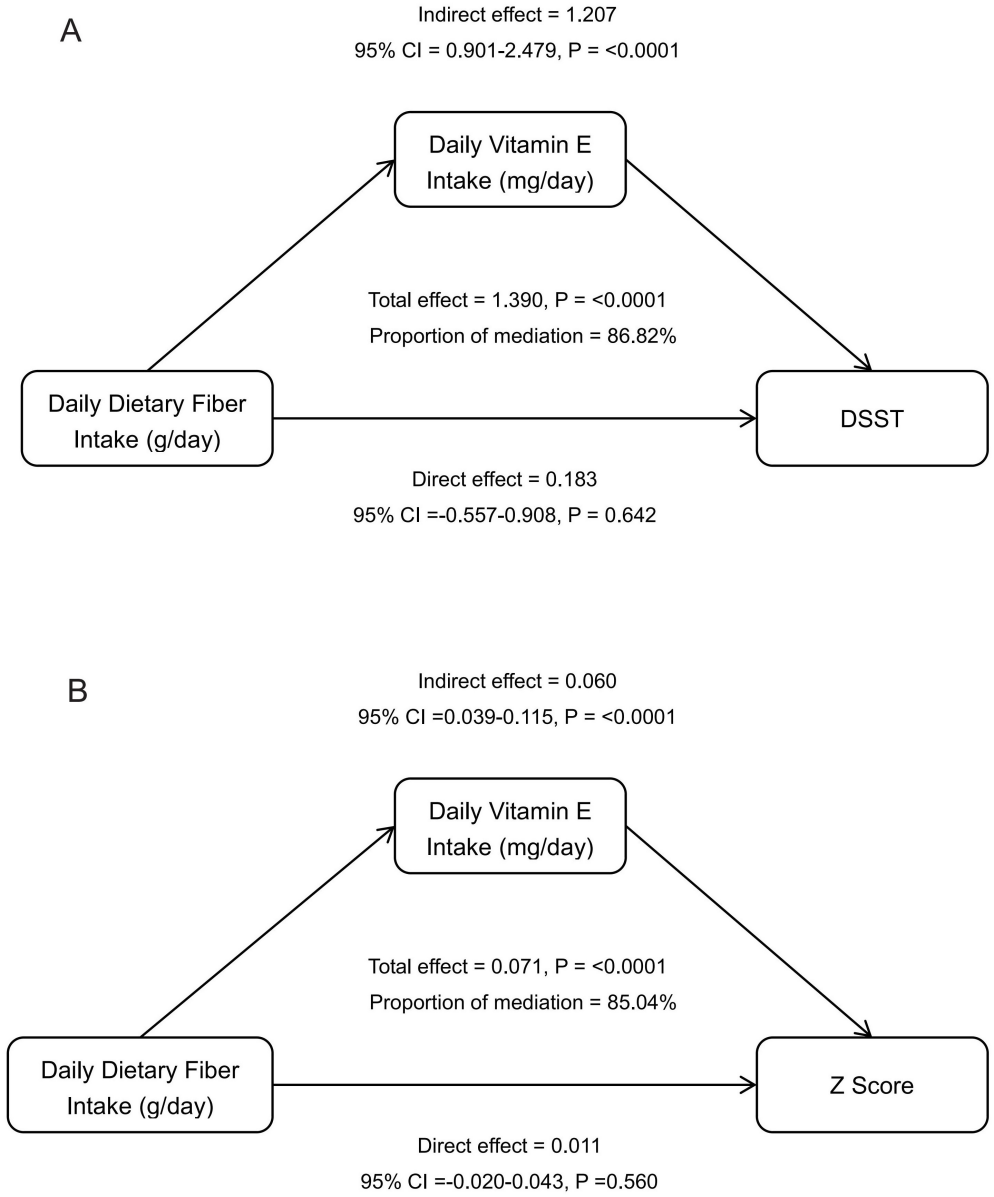
Mediation analysis of vitamin E intake in the relationship between dietary fiber intake and cognitive performance. Path diagrams illustrating the mediating role of daily vitamin E intake in the relationship between dietary fiber intake and various cognitive outcome measures: **(A)** Digit Symbol Substitution Test (DSST), and **(B)** Composite *z*-score. All models were adjusted for demographic and health-related covariates including gender, age, race, education level, annual family income, alcohol consumption status, hypertension, diabetes, physical activity, depression, and vitamin B1 and D intake levels (*n* = 2,529).

To further explore the underlying mechanisms linking dietary fiber intake with DSST scores and composite cognitive *z*-scores, we conducted mediation analysis. As shown in Table 4 and Figure 3, the indirect effect of dietary fiber on DSST score through vitamin E intake was 0.102 (95% CI: 0.062, 0.146; *P* < 0.0001), and for the composite cognitive *z*-score, the indirect effect was 0.098 (95% CI: 0.059, 0.143; *P* < 0.0001). The proportion of the total effect mediated by vitamin E was 86.82% for DSST (95% CI: 55.85%, 160.29%; *P* < 0.0001) and 85.04% for the *z*-score (95% CI: 54.94%, 140.62%; *P* < 0.0001). Subgroup analyses further indicated that this mediating effect remained significant across strata of gender (male, female), hypertension (yes or no), diabetes (yes or no), and among participants without depression. However, the mediation effect did not reach statistical significance in participants with depression or borderline diabetes, which is likely attributable to the limited sample size in these subgroups (depression: *n* = 225; borderline diabetes: *n* = 118).

**Table 4:** Mediation analysis of vitamin E intake in the relationship between dietary fiber intake and cognitive performance across different subgroups.

**Subgroup**	**Sample size**	**DSST**				***Z*** **-score**			
		**Percentage mediated**	**95% CI Lower**	**95% CI Upper**	***P*** **-value**	**Percentage mediated**	**95% CI Lower**	**95% CI Upper**	***P*** **-value**
Total	2,562	86.82	55.85	160.29	<0.0001	85.04	54.94	140.62	<0.0001
**Gender**									
Male	1,253	69.81	32.74	162.64	0.0020	60.92	32.28	119.52	<0.0001
Female	1,309	118.76	53.57	357.58	0.0240	126.24	44.16	641.04	0.0340
**Hypertension status**									
Without hypertension	958	70.18	31.36	180.26	0.0020	54.12	20.74	111.89	0.0020
Hypertension	1,604	90.24	42.52	317.99	0.0100	113.85	48.55	410.15	0.0240
**Depression status**									
Without depression	2,337	85.20	52.65	167.87	<0.0001	97.16	59.31	182.04	0.0020
Depression	225	484.91	−1,560.00	1,374.00	0.5520	17.96	−57.93	203.15	0.2660
**Diabetes status**									
Diabetes	594	117.13	56.65	447.41	0.0240	162.60	64.33	790.43	0.042
Without diabetes	1,850	70.90	39.91	136.62	<0.0001	57.80	28.59	110.17	<0.0001
Borderline	118	163.00	−668.48	887.45	0.3960	115.00	−372.25	534.15	0.4440

DSST, Digit Symbol Substitution Test; z-score, Composite cognitive score.

Model adjustments: All models were adjusted for gender, age, race, education level, annual family income, alcohol status, hypertension, diabetes, physical activity, depression, vitamin B1 intake, and vitamin D intake. In each case, the model is not adjusted for the stratification variable.

## 4 Discussion

Through analysis of data from 2,713 older adults aged ≥60 years in the NHANES 2011–2014 database, there appears to be J-shaped relationships between dietary fiber intake and both DSST and total cognitive scores, suggesting the existence of optimal intake ranges. Below the inflection points (29.65 g/day for DSST and 22.65 g/day for total cognitive scores), increases in dietary fiber intake associated with improved cognitive function. However, beyond these thresholds, this beneficial relationship appeared to attenuate or disappear. Notably, these thresholds generally align with current dietary guidelines recommending a fiber intake of ~25–30 g/day, although actual intake in the population typically falls substantially below these recommended levels (6). Furthermore, our mediation analysis revealed that vitamin E intake partially mediated the relationship between dietary fiber intake and cognitive function, highlighting a potential pathway through which dietary fiber may benefit cognitive health.

Four previous studies based on NHANES 2011–2014 data have explored the association between dietary fiber intake and cognitive function in older adults. For starters, Prokopidis et al. (5) analyzed 1,070 adults aged ≥60 years and found a significant positive correlation between dietary fiber intake (mean 17.3 g/day) and DSST performance, with cognitive benefits plateauing at ~34 g/day. Moreover, Zhang et al. (6) examined 2,478 older adults and revealed that uncontrolled hypertension combined with low dietary fiber intake increased the risk of cognitive impairment, while high fiber intake appeared to mitigate the negative effects of hypertension on cognitive function. In addition, Li et al. (4) investigated 2,461 older patients with chronic kidney disease, demonstrating that those with low fiber intake ( ≤ 25 g/day) performed worse on CERAD-word list and DSST tests, and higher fiber intake seemed to reduce the risk of cognitive impairment in chronic kidney disease patients. Finally, Liang et al. (22) studied 2,189 older adults, confirming the association between chronic obstructive pulmonary disease (COPD) and reduced learning ability and processing speed, but found no significant moderation effect of dietary fiber on this relationship. Our study builds on this by identifying a J-shaped relationship between dietary fiber intake and cognitive function. Cognitive benefits were most pronounced at the inflection points of 29.65 g/day for DSST scores and 22.65 g/day for overall cognitive scores, with each 1 g/day increase in fiber intake associated with measurable cognitive enhancement effects (DSST: 0.18 points per 1 g/day increase in fiber intake, 95% CI: 0.01–0.26; overall cognitive score: 0.01 points per 1 g/day increase, 95% CI: 0.00–0.01). These findings aligns with Prokopidis et al.'s (5) observation of cognitive function plateauing at 34 g/day fiber intake and our study complements this by offering a detailed quantitative analysis. The lack of significant moderation effect of dietary fiber in the COPD study (22) may be attributed to methodological differences or disease-specific factors of the dietary fiber-cognitive function relationship.

Our findings demonstrate that vitamin E plays a significant mediating role in the relationship between dietary fiber and cognitive function, accounting for 85.0% of the total effect. This finding suggests that dietary fiber may influence cognitive function by modulating vitamin E absorption, metabolism, or bioavailability. Vitamin E, as a potent antioxidant, possesses the capacity to attenuate and reduce oxidative stress and neuroinflammation (23), which are known pathological mechanisms leading to cognitive decline (24). Research indicates that higher plasma vitamin E levels are associated with better cognitive performance in older adults and individuals with mild cognitive impairment (25, 26). Furthermore, fiber-rich foods are frequently abundant in antioxidant components which includes vitamin E, providing a theoretical foundation for its substantial mediating effect (27). Several studies have also demonstrated that dietary fiber sources such as whole grains, nuts, seeds, and fruits not only supply vitamin E but also contain other nutrients and bioactive compounds that synergistically enhance brain health (28, 29).

We speculate that dietary fiber may influence cognitive function through multiple potential mechanisms. One primary mechanism is that dietary fiber enhances gut microbiota diversity and promotes the production of short-chain fatty acids (SCFAs), which transmit signals through the gut-brain axis, thereby modulating neuroinflammation and cognitive function. Indeed, SCFAs produced by gut microbiota plays a vital role for maintaining intestinal health and promoting neuronal growth and repair (30, 31). Additionally, dietary fiber attenuates glucose absorption by reducing glycemic fluctuations, and this glycemic stability positively influences cognitive performance. Previous studies have identified direct associations between frequent glycemic excursions and cognitive deficits, particularly in elderly populations (32, 33). Finally, dietary fiber improves lipid profiles and blood pressure regulation, not only promoting cardiovascular health but also providing indirect neuroprotection. These connections between cardiac and cerebral health have received increasing attention, with research demonstrating that poor cardiovascular health may exert potential negative effects on cognitive deterioration (34).

Our research findings suggest that dietary fiber has a significant impact on DSST scores, which primarily assess cognitive functions such as processing speed and working memory, which requires the integrity of specific neural circuits. The prefrontal cortex, parietal lobe, and white matter connections are crucial for processing speed and working memory, and evidence indicates that dietary fiber may provide protective effects on the integrity of white matter (28). In contrast, other cognitive functions like semantic fluency and various memory types tend to rely more on temporal lobe structures and hippocampal networks (29). Additionally, the mechanisms by which dietary fiber can specifically enhance DSST scores may relate to its protective properties against oxidative stress and neuroinflammation—conditions known to negatively impact cognitive functioning. Numerous studies have shown that dietary fiber possesses antioxidant and anti-inflammatory properties that may help mitigate these detrimental effects (31, 35). Moreover, it is crucial to acknowledge the role of the gut microbiota in this context as certain dietary fibers promote the growth of beneficial bacteria that produce precursors for neurotransmitters such as tryptophan and tyrosine, potentially influencing systems relevant to processing speed to a greater extent than those related to memory (36). Notably, the association between fiber intake and cognitive capabilities, particularly in the aging population, hints at the complex interplay between dietary habits and neurological health, suggesting that higher fiber consumption correlates with elevated cognitive test scores, including those from the DSST (5, 6).

However, excessive dietary fiber intake has been associated with various adverse physiological effects, particularly when consumption markedly exceeds recommended levels or when intake is rapidly increased. Very high fiber consumption may lead to gastrointestinal symptoms such as bloating, flatulence, diarrhea, abdominal discomfort, and in severe cases, intestinal obstruction or phytobezoar formation (37, 38). Furthermore, excess fiber can interfere with the absorption of essential minerals—including calcium, magnesium, zinc, and copper—by forming insoluble complexes or reducing transit time, potentially resulting in deficiencies (38, 39). Additionally, sudden or unbalanced increases in fiber may disrupt protein and energy metabolism and adversely alter the gut microbiota composition (39, 40). Consistent with these findings, our study observed that the positive association between dietary fiber intake and global cognitive function diminished when fiber intake surpassed a certain threshold. Notably, an excessive fiber intake was associated with a decline in DSST scores, suggesting potential adverse effects on specific cognitive domains. These results underscore the importance of maintaining an appropriate range of dietary fiber intake.

Several limitations should be acknowledged. First, the cross-sectional design precludes causal inference between dietary fiber intake and cognitive outcomes. Second, dietary data were self-reported, introducing potential recall bias and measurement error. Although multiple confounders were adjusted for, residual confounding remains possible. Third, our analysis did not distinguish between soluble and insoluble fiber types, which may differentially influence cognitive function. Fourth, cognitive assessments were conducted at a single time point; longitudinal studies with repeated measures are needed to examine temporal relationships. Methodologically, while non-linear modeling provides flexibility, curve-fitting approaches carry risks of overfitting, and threshold effects may yield sample-dependent cut-off points lacking biological plausibility. Furthermore, generalizability may be limited as our findings derive from a population from the United States of America with distinct dietary and sociocultural contexts. Finally, NHANES lacks clinical cognitive diagnoses; cognitive status was assessed by neuropsychological tests, precluding identification of specific disorders and possibly limiting generalizability.

## 5 Conclusion

Our study revealed J-shaped relationships between dietary fiber intake and cognitive function, with benefits observed up to thresholds of 29.65 g/day for DSST and 22.65 g/day for total cognitive scores, which align with current dietary recommendations. In addition, vitamin E significantly mediated this relationship, suggesting a potential mechanism through which dietary fiber may support cognitive health in older adults.

## Data Availability

The datasets analyzed for this study can be found in the National Health and Nutrition Examination Survey (NHANES). The data can be accessed through the Centers for Disease Control and Prevention website at https://wwwn.cdc.gov/nchs/nhanes/continuousnhanes/default.aspx?BeginYear=2011 and https://wwwn.cdc.gov/nchs/nhanes/continuousnhanes/default.aspx?BeginYear=2013.
