# Supplementary material for: Non-linear association between dietary fiber intake and cognitive function mediated by vitamin E: a cross-sectional study in older adults
Source: Front Nutr. 2025 Jul 2;12:1611162. doi: 10.3389/fnut.2025.1611162 (PMC12263355; doi:10.3389/fnut.2025.1611162)
Supplement: Supplementary file 1 [file Table_1.docx]

**Supplementary table 1: Evaluation of Covariate Multicollinearity in Cognitive Assessment and Dietary Fiber: Variance inflation factors (VIF) Results**

| **Variables** | **CERAD.IRT** | **CERAD.DRT** | **AFT** | **DSST** | **Z score** | **Dietary Fiber** |
| --- | --- | --- | --- | --- | --- | --- |
| Dietary fiber (g/day) | 2.9 | 2.9 | 2.9 | 2.9 | 2.9 | — |
| Age (years) | 1.2 | 1.2 | 1.2 | 1.2 | 1.2 | 1.2 |
| Gender(Male/Female) | 1.7 | 1.7 | 1.7 | 1.7 | 1.7 | 1.7 |
| Race/ethnicity (categorical) | 1.1 | 1.1 | 1.1 | 1.1 | 1.1 | 1.1 |
| Education level (years) | 1.3 | 1.3 | 1.3 | 1.3 | 1.3 | 1.3 |
| Marital status (categorical) | 1.2 | 1.2 | 1.2 | 1.2 | 1.2 | 1.2 |
| Annual family income (categorical) | 1.3 | 1.3 | 1.3 | 1.3 | 1.3 | 1.3 |
| Alcohol consumption (categorical) | 1.2 | 1.2 | 1.2 | 1.2 | 1.2 | 1.2 |
| Smoking status (categorical) | 1.2 | 1.2 | 1.2 | 1.2 | 1.2 | 1.2 |
| Physical activity (categorical) | 1.1 | 1.1 | 1.1 | 1.1 | 1.1 | 1.1 |
| Hypertension (Yes/No) | 1.1 | 1.1 | 1.1 | 1.1 | 1.1 | 1.1 |
| Diabetes (Yes/No) | 1.1 | 1.1 | 1.1 | 1.1 | 1.1 | 1.1 |
| Depression (Yes/No) | 1.1 | 1.1 | 1.1 | 1.1 | 1.1 | 1.1 |
| Body mass index, BMI (kg/m²) | 6.0 | 6.0 | 6.0 | 6.0 | 6.0 | 6.0 |
| Waist circumference (cm) | 6.2 | 6.2 | 6.2 | 6.2 | 6.2 | 6.2 |
| **Dietary intake** |  |  |  |  |  |  |
| Energy, kcal/d | 45.8 | 45.8 | 45.8 | 45.8 | 43.3 | 43.3 |
| Protein, g/d | 4.8 | 4.8 | 4.8 | 4.8 | 4.6 | 4.6 |
| Fat, g/d | 11.7 | 11.7 | 11.7 | 11.7 | 11.4 | 11.4 |
| Carbohydrate, g/d | 27.8 | 27.8 | 27.8 | 27.8 | 21.2 | 21.2 |
| Sugars, g/d | 6.3 | 6.3 | 6.3 | 6.3 | 5.1 | 5.1 |
| Vitamin B1, mg/d | 3.5 | 3.5 | 3.5 | 3.5 | 3.5 | 3.5 |
| Vitamin B2, mg/d | 3.3 | 3.3 | 3.3 | 3.3 | 3.3 | 3.3 |
| Vitamin B6, mg/d | 2.4 | 2.4 | 2.4 | 2.4 | 2.4 | 2.4 |
| Vitamin B12, μg/d | 1.7 | 1.7 | 1.7 | 1.7 | 1.6 | 1.6 |
| Vitamin C, mg/d | 1.6 | 1.6 | 1.6 | 1.6 | 1.5 | 1.5 |
| Vitamin D, μg/d | 1.5 | 1.5 | 1.5 | 1.5 | 1.5 | 1.5 |
| Vitamin E, mg/d | 2.4 | 2.4 | 2.4 | 2.4 | 2.2 | 2.2 |

**Abbreviations**: VIF=Variance inflation factors; BMI=body mass index; CERAD=Consortium to Establish a Registry for Alzheimer's Disease; IRT=Item Response Theory; DRT=Delayed Recall Test; AFT =Animal Fluency Test; DSST=Digit Symbol Substitution Test; Z-score =Standardized composite cognitive score
