# Supplementary material for: Non-linear association between dietary fiber intake and cognitive function mediated by vitamin E: a cross-sectional study in older adults
Source: Front Nutr. 2025 Jul 2;12:1611162. doi: 10.3389/fnut.2025.1611162 (PMC12263355; doi:10.3389/fnut.2025.1611162)
Supplement: Supplementary file 2 [file Table_2.docx]

**Supplementary Table 2：Threshold Effect of Dietary Fiber Intake on DSST Scores Stratified by Gender**

| **Outcome** | **Male**  **β (95% CI)** | **P-value** | **Female**  **β (95% CI)** | **P-value** | **P-interaction** |
| --- | --- | --- | --- | --- | --- |
| Model I |  |  |  |  | 0.909 |
| One line effect | 0.09 (0.01, 0.17) | 0.0203 | 0.04 (−0.07, 0.15) | 0.4452 |  |
| Model II |  |  |  |  | 0.497 |
| Turning Point (K) | 9.85 | – | 21.05 | – |  |
| Dietary fiber intake < K | 1.05 (0.53, 1.57) | <0.0001 | 0.19 (0.03, 0.36) | 0.0316 |  |
| Dietary fiber intake ≧ K | 0.05 (-0.04, 0.13) | 0.2667 | -0.16 (-0.36, 0.04) | 0.1204 |  |
| P value for LRT test | – | <0.001 | – | 0.018 |  |
| 95% CI for tuning point | 41.92 - 44.57 | – | 51.51 - 55.04 | – |  |

**Note:** DSST = Digit Symbol Substitution Test; LRT = logarithm likelihood ratio test. Model I represents linear regression analysis; Model II represents curve-fitting threshold effect analysis. All models were adjusted for age, race, education level, annual family income, alcohol status, hypertension, diabetes, physical activity, depression, vitamin B1 intake, and vitamin D intake.
