# Supplementary material for: Non-linear association between dietary fiber intake and cognitive function mediated by vitamin E: a cross-sectional study in older adults
Source: Front Nutr. 2025 Jul 2;12:1611162. doi: 10.3389/fnut.2025.1611162 (PMC12263355; doi:10.3389/fnut.2025.1611162)
Supplement: Supplementary file 3 [file Table_3.docx]

**Supplementary Table 3：Threshold Effect of Dietary Fiber Intake on DSST Scores Stratified by Hypertension Status**

| **Outcome** | **Without Hypertension**  **β (95% CI)** | **P-value** | **Hypertension**  **β (95% CI)** | **P-value** | **P-interaction** |
| --- | --- | --- | --- | --- | --- |
| Model I |  |  |  |  | 0.496 |
| One line effect | 0.09 (0.00, 0.18) | 0.0502 | 0.08 (-0.02, 0.17) | 0.1155 |  |
| Model II |  |  |  |  | 0.720 |
| Turning Point (K) | 5.7 | – | 12.55 | – |  |
| Dietary fiber intake < K | 1.73 (0.37, 3.09) | 0.0125 | 0.80 (0.41, 1.18) | <0.0001 |  |
| Dietary fiber intake ≧ K | 0.07 (-0.02, 0.16) | 0.1381 | -0.00 (-0.11, 0.10) | 0.9448 |  |
| P value for LRT test | – | 0.017 | – | <0.001 |  |
| 95% CI for tuning point | 41.64 - 44.41 | – | 49.00 - 52.14 | – |  |

**Note:** DSST = Digit Symbol Substitution Test; LRT = logarithm likelihood ratio test. Model I represents linear regression analysis; Model II represents curve-fitting threshold effect analysis. All models were adjusted for gender, age, race, education level, annual family income, alcohol status, diabetes, physical activity, depression, vitamin B1 intake, and vitamin D intake.
