# Supplementary material for: Non-linear association between dietary fiber intake and cognitive function mediated by vitamin E: a cross-sectional study in older adults
Source: Front Nutr. 2025 Jul 2;12:1611162. doi: 10.3389/fnut.2025.1611162 (PMC12263355; doi:10.3389/fnut.2025.1611162)
Supplement: Supplementary file 4 [file Table_4.docx]

**Supplementary Table 4：Threshold Effect of Dietary Fiber Intake on DSST Scores Stratified by Depression Status**

| **Outcome** | **Without Depression**  **β (95% CI)** | **P-value** | **Depression**  **β (95% CI)** | **P-value** | **P-interaction** |
| --- | --- | --- | --- | --- | --- |
| Model I |  |  |  |  | 0.786 |
| One line effect | 0.08 (0.01, 0.15) | 0.0236 | 0.01 (−0.22, 0.24) | 0.9541 |  |
| Model II |  |  |  |  | 0.834 |
| Turning Point (K) | 29.7 | – | 12.5 | – |  |
| Dietary fiber intake < K | 0.15 (0.09, 0.22) | <0.0001 | 0.10 (−0.07, 0.27) | 0.255 |  |
| Dietary fiber intake ≧ K | 0.02 (−0.09, 0.12) | 0.748 | 0.06 (−0.11, 0.23) | 0.465 |  |
| P value for LRT test | – | 0.002 | – | 0.464 |  |
| 95% CI for tuning point | 20.5 - 54.2 | – | 3.1 - 45.4 | – |  |

**Note:** DSST = Digit Symbol Substitution Test; LRT = logarithm likelihood ratio test. Model I represents linear regression analysis; Model II represents curve-fitting threshold effect analysis. All models were adjusted for gender, age, race, education level, annual family income, alcohol status, hypertension, diabetes, physical activity, vitamin B1 intake, and vitamin D intake.
