# Supplementary material for: Non-linear association between dietary fiber intake and cognitive function mediated by vitamin E: a cross-sectional study in older adults
Source: Front Nutr. 2025 Jul 2;12:1611162. doi: 10.3389/fnut.2025.1611162 (PMC12263355; doi:10.3389/fnut.2025.1611162)
Supplement: Supplementary file 5 [file Table_5.docx]

**Supplementary Table 5：Threshold Effect of Dietary Fiber Intake on DSST Scores Stratified by Diabetes Status**

| **Outcome** | **Diabetes**  **β (95% CI)** | **P-value** | **Without Diabetes β (95% CI)** | **P-value** | **Borderline**  **β (95% CI)** | **P-value** | **P-interaction** |
| --- | --- | --- | --- | --- | --- | --- | --- |
| Model I |  |  |  |  |  |  | 0.713 |
| One line effect | 0.05 (-0.09, 0.19) | 0.5240 | 0.09 (0.01, 0.16) | 0.0262 | 0.19 (-0.13, 0.50) | 0.2447 |  |
| Model II |  |  |  |  |  |  | 0.694 |
| Turning Point (K) | 7.7 | – | 33.3 | – | 8.35 | – |  |
| Dietary fiber intake < K | 1.94 (0.90, 2.98) | 0.0003 | 0.19 (0.09, 0.28) | 0.0001 | 2.41 (-0.00,4.83) | 0.0529 |  |
| Dietary fiber intake ≧ K | -0.03 (-0.18, 0.11) | 0.6680 | -0.19 (-0.36, -0.02) | 0.0298 | 0.09 (-0.23, 0.42) | 0.5719 |  |
| P value for LRT test | – | <0.001 | – | <0.001 | – | 0.047 |  |
| 95% CI for tuning point | 39.18 - 43.27 | – | 52.66 - 56.59 | – | 43.09 - 52.45 | – |  |

**Note:** DSST = Digit Symbol Substitution Test; LRT = logarithm likelihood ratio test. Model I represents linear regression analysis; Model II represents curve-fitting threshold effect analysis. All models were adjusted for gender, age, race, education level, annual family income, alcohol status, hypertension, physical activity, depression, vitamin B1 intake, and vitamin D intake.
