# Supplementary material for: Non-linear association between dietary fiber intake and cognitive function mediated by vitamin E: a cross-sectional study in older adults
Source: Front Nutr. 2025 Jul 2;12:1611162. doi: 10.3389/fnut.2025.1611162 (PMC12263355; doi:10.3389/fnut.2025.1611162)
Supplement: Supplementary file 6 [file Table_6.docx]

**Supplementary Table 6：Threshold Effect of Dietary Fiber Intake on Z Scores Stratified by Gender**

| **Outcome** | **Male**  **β (95% CI)** | **P-value** | **Female**  **β (95% CI)** | **P-value** | **P-interaction** |
| --- | --- | --- | --- | --- | --- |
| Model I |  |  |  |  | 0.709 |
| One line effect | 0.00 (0.00, 0.01) | 0.0153 | 0.00 (-0.00, 0.01) | 0.1739 |  |
| Model II |  |  |  |  | 0.467 |
| Turning Point (K) | 7.55 | – | 13.6 | – |  |
| Dietary fiber intake < K | 0.07 (0.03, 0.12) | 0.0016 | 0.02 (0.01, 0.03) | 0.0077 |  |
| Dietary fiber intake ≧ K | 0.00 (-0.00, 0.01) | 0.0851 | 0.00 (-0.01, 0.01) | 0.7883 |  |
| P value for LRT test | – | 0.003 | – | 0.019 |  |
| 95% CI for tuning point | -0.25 - -0.13 | – | 0.15 - 0.28 | – |  |

**Note:** LRT= logarithm likelihood ratio test; Z-score = standardized composite cognitive score. Model I represents linear regression analysis; Model II represents curve-fitting threshold effect analysis. All models were adjusted for age, race, education level, annual family income, alcohol status, hypertension, diabetes, physical activity, depression, vitamin B1 intake, and vitamin D intake.
