# Supplementary material for: Non-linear association between dietary fiber intake and cognitive function mediated by vitamin E: a cross-sectional study in older adults
Source: Front Nutr. 2025 Jul 2;12:1611162. doi: 10.3389/fnut.2025.1611162 (PMC12263355; doi:10.3389/fnut.2025.1611162)
Supplement: Supplementary file 8 [file Table_8.docx]

**Supplementary Table 8：Threshold Effect of Dietary Fiber Intake on Z Scores Stratified by Depression Status**

| **Outcome** | **Without Depression**  **β (95% CI)** | **P-value** | **Depression**  **β (95% CI)** | **P-value** | **P-interaction** |
| --- | --- | --- | --- | --- | --- |
| Model I |  |  |  |  | 0.029 |
| One line effect | 0.00 (0.00, 0.01) | 0.0236 | 0.01 (-0.00, 0.02) | 0.1515 |  |
| Model II |  |  |  |  | 0.144 |
| Turning Point (K) | 12.2 | – | 4.7 | – |  |
| Dietary fiber intake < K | 0.02 (0.01, 0.03) | 0.0023 | 0.20 (0.03, 0.37) | 0.0239 |  |
| Dietary fiber intake ≧ K | 0.00 (-0.00, 0.01) | 0.3577 | 0.01 (-0.01, 0.02) | 0.3473 |  |
| P value for LRT test | – | 0.010 | – | 0.023 |  |
| 95% CI for tuning point | 0.04 - 0.13 | – | -0.52 - -0.20 | – |  |

**Note:**  LRT = logarithm likelihood ratio test; Z-score = standardized composite cognitive score. Model I represents linear regression analysis; Model II represents curve-fitting threshold effect analysis. All models were adjusted for gender, age, race, education level, annual family income, alcohol status, hypertension, diabetes, physical activity, vitamin B1 intake, and vitamin D intake.
