# Supplementary material for: Non-linear association between dietary fiber intake and cognitive function mediated by vitamin E: a cross-sectional study in older adults
Source: Front Nutr. 2025 Jul 2;12:1611162. doi: 10.3389/fnut.2025.1611162 (PMC12263355; doi:10.3389/fnut.2025.1611162)
Supplement: Supplementary file 9 [file Table_9.docx]

**Supplementary Table 9：Threshold Effect of Dietary Fiber Intake on Z Scores Stratified by Diabetes Status**

| **Outcome** | **Diabetes**  **β (95% CI)** | **P-value** | **Without Diabetes β (95% CI)** | **P-value** | **Borderline**  **β (95% CI)** | **P-value** | **P-interaction** |
| --- | --- | --- | --- | --- | --- | --- | --- |
| Model I |  |  |  |  |  |  | 0.585 |
| One line effect | 0.00 (-0.01, 0.01) | 0.7727 | 0.01 (0.00, 0.01) | 0.0018 | 0.00 (-0.02, 0.02) | 0.7552 |  |
| Model II |  |  |  |  |  |  | 0.501 |
| Turning Point (K) | 7.65 | – | 13.05 | – | 29.25 | – |  |
| Dietary fiber intake < K | 0.07 (0.02, 0.13) | 0.0089 | 0.02 (0.01, 0.03) | 0.0010 | 0.01 (-0.01, 0.03) | 0.4761 |  |
| Dietary fiber intake ≧ K | -0.00 (-0.01, 0.01) | 0.6375 | 00 (-0.00, 0.01) | 0.1154 | 0.04 (-0.11, 0.04) | 0.3508 |  |
| P value for LRT test | – | 0.008 | – | 0.011 | – | 0.253 |  |
| 95% CI for tuning point | 0.25 - -0.07 | – | 0.09 - 0.19 | – | 0.22 - 0.44 | – |  |

**Note:**  LRT = logarithm likelihood ratio test; Z-score = standardized composite cognitive score. Model I represents linear regression analysis; Model II represents curve-fitting threshold effect analysis. All models were adjusted for gender, age, race, education level, annual family income, alcohol status, hypertension, physical activity, depression, vitamin B1 intake, and vitamin D intake.
